# How do mentors perceive and perform their role in a reflection-based mentoring programme for medical students?

**DOI:** 10.5116/ijme.6712.35da

**Published:** 2024-10-28

**Authors:** Fleur Helewaut, Jan Reniers, Ellen Paelinck, Serhat Yildirim

**Affiliations:** 1 Clinical Skills Training Centre, Faculty of Medicine and Health Sciences, Ghent University, Belgium; 2 Faculty of Medicine and Health Sciences, Ghent University, Belgium

**Keywords:** Medical students, mentoring, mentor profiles, reflection

## Abstract

**Objectives:**

To explore how mentors perceive and perform their role in a longitudinal mentorship programme with the objective of guiding medical students in becoming reflective learners.

**Methods:**

A qualitative exploratory study was conducted using semi-structured interviews with 16 mentors from the Ghent University medical education mentoring programme. Participants were selected by purposeful sampling on gender, years of experience and area of specialisation. Interview transcripts were analysed using inductive thematic analysis. Afterwards, all transcripts were re-analysed combining the resulting themes to identify different mentoring profiles.

**Results:**

Our analysis yielded three themes. First, the basic conditions for mentorship showed wide differences in competencies that mentors considered necessary. Second, goals and purposes of mentoring identified roles ranging from ombudsperson, confidential advisor, role model to guide towards professional and personal growth. Third, attitudes to the mentoring programme revealed a wide variation from fully embracing to rejecting the reflective method. Further analysis led to three mentor profiles: reflective, sharing and advising. Even reflective mentors struggled in varying degrees with applying the guidelines, mostly depending on prior experience with reflection. Advising mentors found the intervision techniques too constrictive and expressed doubts about the usefulness of the programme.

**Conclusions:**

In this reflection-based mentoring programme, different mentor perceptions strongly determined how reflection is being taught to medical students. This may affect the students’ professional identity formation. Training should enable mentors to reflect on their beliefs and mentoring style. Further research is needed on the effects of reflection in mentoring and on mentor selection.

## Introduction

Reflection is an essential component of medical education. It refers to the human capacity to look back in a structured way at one’s experiences and actions. This can take place in many ways and can involve thought, experiences, emotions, the body, and others. Reflection is a metacognitive process that creates greater understanding of oneself and situations to steer future action.^1^^-^^4^ It is considered a core activity in professional identity formation (PIF).^5^^,^^6^ Reflection by medical students with role models and mentors leads to a repetitive pattern that begins with the exploration of new knowledge and experiences and results in the learner’s assimilation into an existing identity.^5^ When explicitly encouraged to reflect, learners become active participants in the formation of their own identity.^5^  A review of interventions reporting the use of reflection in graduate medical education suggests that reflection has a positive impact on empathy, increases comfort with learning in complex situations, and enhances engagement in the learning process.^1^ In addition, reflection is an important competency for providing compassionate care. It is necessary to come to an understanding of what will serve the patient and to recognise the influence of one’s own thoughts and emotions when entering into a therapeutic relationship.^7^^-^^9^ Students need support in their professional development, especially with regard to negative role modelling, if they are to avoid the development of attitudes such as othering, distancing, detachment, and dehumanisation.^10^^,^^11^

While reflection is considered an internal process, it can be fostered during collaborative learning.^2^^,^^12^ Guided reflection with a supervisor or mentor is important so that underlying beliefs and assumptions can be challenged within a supportive relationship.^13^ Mentoring’s personalised longitudinal and holistic support helps medical students and physicians in training to internalise the characteristics, values, and norms of the medical profession, resulting in individual thinking, acting, and feeling like a physician.^14^  In this way mentoring plays a critical role in nurturing PIF.^15^

However, there is little information on how reflective practices are integrated and stimulated in mentoring programmes. The way individual mentors practice their role is influenced by their personal beliefs and understanding of the goals and purposes of mentoring.^16^^-^^20^ They may differ in their beliefs about what constitute meaningful mentoring activities, who should decide on the focus of the mentoring activity and which strategies and methods should be used to enact these beliefs.^20^ A different understanding of their mentor role will affect their actions and their relationship with the mentees.^19^ Understanding this process is crucial when implementing a mentoring programme, and even more so in longitudinal programmes where it will affect the mentor’s relationship with the students for many years.

The aim of this study is to explore how mentors perceive and perform their roles and try to meet expectations in a longitudinal and structured mentoring programme with the clear objective of guiding medical students in becoming reflective learners.

## Methods

### Study Setting

In Belgium medical students complete a six-year undergraduate and graduate medical curriculum. Since 2012, the Faculty of Medicine and Health Sciences of Ghent University, Belgium, organises a mandatory group mentoring programme as part of integrating professionalism in the medical curriculum. The ultimate goal of this programme is to create continuity in the professional development of the students into reflective practitioners, thereby positively affecting the well-being of both the students and their future patients.

All mentors in the programme are physicians, enabling them to serve as a role model for medical students. The only selection criterion is a commitment to invest time, energy and effort in mentoring students according to the reflective guidelines of the programme.  Every mentor guides nine to ten medical students, both on a group basis and individually during the entire course of their six-year curriculum. After 6 years mentors are invited to start with a new group of students. There are currently 220 mentors active in supporting approximately 2,000 students. The mentoring programme is structured in 4 group sessions and 1 individual meeting per year. During the sessions students reflect on experiences they have during the academic year and on medical-ethical issues and social problems. The reflective guidelines and objectives of these sessions are presented to the mentors in training sessions that take place twice a year. Self-reflection and group reflection are facilitated with the intervision model according to Korthagen.^21^

### Study design

We conducted a qualitative explorative study using individual semi-structured interviews enabling us to explore mentors’ experiences in a longitudinal reflection-based mentoring programme. Interviews were considered an appropriate method to discover mentors’ thoughts, perspectives and experiences regarding their role and performance and the expectations of the mentoring programme.^22^

### Study Participants

All participating mentors were either specialists, working at the Ghent University Hospital, or general practitioners and specialists from the Ghent region. A purposeful sample of 16 mentors was recruited by e-mail invitation. We aimed to include 2 equal groups of mentors: one group with 4 to 6 years of mentoring experience and another with 8 to 10 years of experience. Within both groups we randomly selected 8 men and 8 women. In our final selection of 16 mentors we ensured diversity in area of specialisation. Initially, 13 mentors responded positively and were subsequently contacted to schedule the interview. Additionally, 3 extra mentors were contacted to participate and all 3 consented. Our final sample consisted of 16 mentors, 8 men and 8 women, half of them in their first cycle of mentoring and the other half in their second cycle. There were 3 general practitioners, 5 physicians from surgical disciplines, and 8 physicians from non-surgical disciplines. The average age of the mentors was 51,8 years and the average years of experience as a mentor was 6,9 years. (Table 1)

The participating mentors received written information about the study and information on voluntary participation, confidentiality and data management. An informed consent form was provided in advance for notification and signature. This study was submitted for assessment and received approval from the Ethics Committee of Ghent University Hospital.

### Data Collection

We conducted semi-structured interviews using a set of open-ended questions. The interview guide was developed based on our search of the literature and the characteristics of our mentoring programme: a longitudinal mentor-mentee relationship, group and one-on-one mentoring, a structured programme with predefined topics and focus on reflection in the mentor training. The interview guide was piloted with two non-participating mentors to assess coverage, relevance and clarity.^23^ Revisions were then made before the start of data collection. The final interview guide comprised 4 topics: (i) motivation and expectations, (ii) mentor roles and relationship towards mentees, (iii) mentor competencies and training, and (iv) group mentoring versus one-on-one mentoring. (Appendix) Two trained undergraduate medical students (EP, SY) conducted interviews between February and May 2021. Due to the COVID-19 pandemic, interviews took place online, with webcam. All interviews were audio-recorded, transcribed verbatim, pseudonymized and verified against the original audio file (EP, SY). The interviews lasted 40-60 minutes and were conducted in Dutch.

**Table 1:** Participant characteristics

Participant	Age	Gender	Specialty	Years of mentoring experience	Mentor cycle
1	57	F	Palliative Care	4	1
2	43	M	Surgery	6	1
3	42	M	Surgery	5	1
4	53	F	Internal Medicine	10	2
5	57	F	Intensive Care	5	1
6	37	F	Internal Medicine	4	1
7	48	M	Surgery	10	2
8	56	M	Intensive Care	8	2
9	50	F	Surgery	9	2
10	40	M	Psychiatry	4	1
11	66	M	Primary Care	9	2
12	51	F	Primary Care	5	1
13	52	F	Internal Medicine	9	2
14	55	F	Internal Medicine	9	2
15	61	M	Primary Care	5	1
16	61	M	Surgery	8	2

### Data Analysis

In a first step, the data were analysed using inductive thematic analysis. This qualitative method aims to identify, analyse, and report patterns within data. We applied the approach to thematic analysis by Virginia Braun and Victoria Clarke, which entails 6 phases: (i) familiarising yourself with the data, (ii) generating initial codes, (iii) searching for themes, (iv) reviewing themes, (v) defining and naming themes and (vi) producing the report.^24^ All authors read all transcripts to familiarize themselves with the data. Subsequently, the transcripts were loaded into the NVivo software for coding.^25^ Two authors (EP and SY) independently coded all transcripts. The coding was supervised and independently reviewed by FH and JR. The research team met the first time after the first 2 transcripts were coded and then regularly after every 4 coded transcripts to discuss and resolve intercoder disagreements.  Subsequently, related codes were grouped into potential themes and subthemes. The analysis of the last 3 interviews yielded no new themes and the team decided that data-saturation was achieved. A first set of quotes that illustrated the meaning and content of the (sub)themes was identified. Finally, we synthesised narrative descriptions for each (sub)theme.

In a second step, we looked for patterns of how the different themes developed in the narrative of each participant. This analysis resulted in the definition and description of mentor profiles. Regular discussions were held to reach consensus on the different profiles. All authors contributed to the narrative description of the different (sub)themes and profiles. A final selection of verbatim quotes was made to illustrate each of the themes and subthemes, based on their overall representativeness, or to reflect differences of opinions between participants. Due to time constraints, a participant check of our findings was not done.

### Trustworthiness and rigor

Two members of the research team (FH and JR) are physicians, mentors and coordinators of the mentoring programme. Both are experienced in medical teaching and intervision and have extensive knowledge about the origin and shaping of the mentoring programme. They are also responsible for the programme’s content development and implementation. One of the authors (FH) has experience in qualitative research and trained the students for the interviewing and coding. The two trained undergraduate students (EP and SY) were mentees in their fifth year without any previous history with the 16 mentors. Their familiarity with the mentoring programme helped them to encourage mentors to illustrate their answers.

To promote reflexivity, we met regularly to discuss and adjust codes and to discuss in-depth the findings from the transcripts and the coding. This was an iterative process of going back and forth between the codes and the original data. We jointly reviewed the themes to ensure that the codes in each (sub)theme were coherent (internal homogeneity), that the codes in different (sub)themes could be clearly distinguished (external heterogeneity) and that themes reflected the coded data and the data set as a whole.^24^

Additionally, in-depth conversations were held to examine our different roles in the mentoring programme and the possible biases in the different phases of the study.^26^ Since two members of the research team are responsible for the development and implementation of the programme, this could create a bias in the analysis of the data. Hence, the students were explicitly invited to critically question assumptions of the two coordinators and vice versa. Notes of all meetings were made and consulted during the process.

## Results

Our analysis yielded three themes. The first theme has to do with basic conditions for mentorship, the second with the goals and purposes mentors deemed important and the third with their attitude towards the mentoring programme.

### Theme: Basic conditions for mentorship

Within this theme two subthemes emerged: the importance of respect and trust and the competencies needed to be a mentor.

#### Respect and trust

The primary conditions for successful mentoring mentioned by almost all mentors were respect and trust. They wanted to create a group where safety and trust are present and where mentees feel confident to participate. Mentees were invited to use the sessions as a time for ventilation and to share their concerns in the group.

“There should always be respect. That's a very important item. There should also be trust... Respect, trust and integrity for what everyone says.”  (Interview 16, male, surgery, second cycle)

“I hope that for most students it's a kind of safe haven anyway, to be different from medical students occupied with knowledge and skills.” (Interview 1, female, palliative care, first cycle)

### Mentor competencies

In the competencies deemed necessary to be a mentor there was a wide variation from being open, curious and enthusiastic, over being empathic and having a clear vision on the medical profession, to being able to self-reflect and learning others to reflect.

“I think it is important that you show enthusiasm and that you also try to explain why it is important what we do. And believing in it yourself, of course.” (Interview 8, male, intensive care, second cycle)

“To be very curious, very modest. Also, as a mentor to be self-critical, reflective, to understand that these are ten young people, ten future colleagues. Let their ideas surprise you, let them inspire you, and dare to question yourself based on what you hear from them.” (Interview 10, male, psychiatry, first cycle)

“You also have to like doing it. I think it really shouldn't be a task, you have to like doing it.” (Interview 5, female, intensive care, first cycle)

“On the one hand you must be critical, be a good listener, be empathic, pay attention, be open. I also think, yes, having a strong vision on what your profession is, and what you think is important, and to dare to stand for that.” (Interview 14, female, internal medicine, second cycle)

“I think you have to be able to teach them especially well how you are going to reflect, how you are going to look at yourself, how you can interpret and explain your own behaviour.” (Interview 9, female, surgery, second cycle)

### Theme: Goals and purposes of mentoring

Within this theme two subthemes emerged: what mentors considered to be their role and what they expected as the outcome. Although the mentoring programme has specific goals and purposes, mentors often had their own perception of their role.

#### Mentor roles

A first role was being an advisor and ombudsperson when students had questions. The perception of this role as the most important one sometimes raised doubts about the usefulness of the mentoring programme.

“I do sincerely wonder sometimes if that (the mentoring programme) has such a great benefit. Maybe that benefit is there if there are problems for a particular student... I think mainly for problems related to the personal interpretation of their studies: the feeling around it, problems they may experience on a personal level that interfere with the study, problems around exams, problems with professors, problems with themselves.” (Interview 7, male, surgery, second cycle)

A second one was serving as a confidential advisor for mentees even outside of the mentor sessions.

“But for other practical problems, around their medical training, but also personal problems… you do indeed need to build up a certain relationship of accessibility and trust.” (Interview 12, female, primary care, first cycle)

A third role was to serve as a role model. Mentors found it important to share what it means to be a doctor and work with patients.

“In my role as mentor, I hope to be a sort of role model. Not as in, I am the great example, but an example because of my experience with patients, students, colleagues, and so on. You can tell the students things about real life. I’m very patient-centred and because respect for the patient is very important to me, I hope, that I can bring this to them.” (Interview 4, female, internal medicine, second cycle)

“The idea is, in my opinion, to bring young people together with more experienced people who are already in the profession and to find a balance in guiding them towards their goal.” (Interview 16, male, surgery, second cycle)

A fourth role was to guide students in their professional and personal growth.

“They learn to reflect, they learn to think about “why did I react this way” or “how would I handle this” or “why does my fellow student react that way”, to be able to empathize and ask in-depth questions.” (Interview 9, female, surgery, second cycle)

“It's called professional development, but I think it's broader than just professional… ultimately, becoming a doctor and being a doctor influences how you are in life, what you find important. Surely that becomes a part of your 'being' and that should not be underestimated.” (Interview 1, female, palliative care, first cycle)

### Expected outcome

The subtheme of expected outcome varied greatly between mentors, from very little expectations to becoming a reflective doctor.

“If, at the end of your six-year journey, you can achieve that they have gained something from it, however small it may be, that is good for me.” (Interview 2, male, surgery, first cycle)

“Being respectful in life, towards patients, also towards colleagues. Then, yes, learning from each other, also listening to each other … and respectfully dealing with each.” (Interview 4, female, internal medicine, second cycle)

“So that reflective ability, the reflective doctor; if all my team-mentees after six years were trained to be very talented doctors with a lot of potential, but especially with a lot of reflective ability, I think I would be very satisfied.” (Interview 10, male, psychiatry, first cycle)

### Theme: Attitude towards the mentoring programme

Within this category three subthemes emerged: attitude towards the script with guidelines, to the reflective method itself and to the mentor training.

### Attitude towards the script with the guidelines

Almost all mentors attached importance to structure during a session, but they did not regard the structured process of a mentor session with the intervision method as self-evident. Some found the script with the topics and guidelines too artificial, constrictive and classroom-like.

“Sometimes, rather strict guidelines on how to perform the mentoring are imposed upon us. But I find it hard to stick to that because I think it is artificial.” (Interview 13, female, internal medicine, second cycle)

It is precisely that classroom-like atmosphere that these mentors hoped to avoid, especially since some students saw the assignments as an extra burden.  They had doubts about the usefulness of the mentoring system.

“Often they consider the topics relatively useless. And that leads me to think: “well, if they find it useless ... I don't have to do it for myself either.” So, you know, you have to weigh the pro versus the con. Their curriculum is so packed that you can wonder if this actually has an added value and if this achieves its goal.” (Interview 7, male, surgery, second cycle)

In contrast, some mentors did find this structure very helpful to support their objectives.

“I think it's much more important that you can teach them to reflect and that you can feel how a mentee works, and at first I thought "yes, that's going to be really hard”, because, all of a sudden you get ten new faces in front of you. But because they make those preparations, and there is a structure provided, you already have an idea whether they go quite deep, or whether they remain superficial. Then you can adjust that during your mentoring sessions.” (Interview 9, female, surgery, second cycle)

Other mentors preferred a looser structure: they started with the guidelines, but then deviated more from the schedule and script, depending on the topic of the sessions.

“Well, I didn't always have a good feeling about that. If you have to work according to that protocol and there is no momentum, it is sometimes very difficult to open it up. And I do notice that, by letting that go a bit, that the students also get a little more confidence in you and that they find themselves less uncomfortable to participate in certain assignments that are given.” (Interview 6, female, internal medicine, first cycle)

### Attitude towards the reflective method

There was a wide variation from rejecting to fully embracing the reflective method, even when this was new to them.

“Yes, now we are going to learn to reflect and then choose a fictive subject .... and yes, that's mandatory. I think a lot of students are going to say: yes, that's because we are obliged to go there, there's not that much added value.” (Interview 7, male, surgery, second cycle)

“I think also being open to the concept of mentoring and of course that mainly involves learning to reflect, that it adds value. I myself didn't think about that before either. What is reflecting? What can it be useful for? Indeed, I do see it as something useful, something to be positive about. Being open to something that did not yet exist in my time.” (Interview 3, male, surgery, first cycle)

“The theoretical model of reflection that the mentoring session is mainly aimed at, is really important; that they learn to reflect, that they learn to think of 'why did I react this way' or 'how would I do that' or 'why does my fellow student react that way'? to empathise with that a little bit and ask in-depth questions there as well.”  (Interview 9, female, surgery, second cycle)

### Attitude towards the mentor training

Some mentors didn’t see the usefulness of the training, while others considered it as essential to keep growing. Many had difficulties to find the time to come to the training.

“I don't think those competencies can be trained, actually. They say "you have to listen, and ...", but yes, we automatically do that already…” (Interview 13, female, internal medicine, second cycle)

“Everything that I think is important in a mentoring session comes up there as well. But the most important thing is of course that you get some insight into "what is the objective of the session, where is the focus, what is the emphasis", so that you know what is expected of you in the session and that you can then work with that yourself.” (Interview 10, male, psychiatry, first cycle)

“I think this training is brilliant and it should not only be given to mentors, but actually to everyone in the hospital. Because you learn there, through the fact that you yourself play that role-play… you actually learn something yourself from that.” (Interview 9, female, surgery, second cycle)

### Mentor profiles

Based on further analysis of the themes and subthemes in the interviews of the sixteen mentors, three mentor profiles could be distinguished. We labelled them reflective, sharing and advising.

### Reflective mentor

Ten ‘reflective’ mentors adhered to the principles and guidelines of the programme. They believe that reflection is the base of the mentoring relationship and expect that their mentees will become ‘reflective’ doctors. They mentioned a wider variety of competencies which they considered necessary for being a mentor and they often described mentoring as a  process of growth. They found the training and the guidelines helpful in providing them with tools that they could use and adapt to their personal way of doing. There is a wide variation in the degree of ease and confidence with which these mentors applied reflection in their sessions. Some clearly struggled with the intervision method in the beginning and gradually became more confident. They learned a lot from each other in the training sessions.

In the beginning, I also had to learn those different points on reflection and questioning. I'm not going to say learn by heart, but I had to get used to it a bit. And then that comes back occasionally over the years. Then you see that you already know that flow well. (Interview 3, male, surgery, first cycle)

And so you hear, you see other mentors, you look at what they are doing, you are sometimes inspired by them …so that is valuable. (Interview 10, male, psychiatry, first cycle)

In further analysis ‘reflective’ mentors were equally divided between men and women (5 each). Six out of 8 first cycle mentors (4 to 6 years of experience) were ‘reflective’ versus 4 out of 8 second cycle mentors (8 to 10 years of experience). Mentors in their second cycle attended the training sessions less frequently because they already knew most of the topics and relied on the scripts with guidelines to refresh their memories. All 3 general practitioners were ‘reflective’ mentors, as were 5 out of 8 internal medicine specialists. Of the 5 surgeons, only 2 were ‘reflective’. One surgeon made this comment:

“Yes, I wasn’t really that prepared for it. I think, if you are a general practitioner or a psychologist, psychiatrist, paediatrician, you really are working on such matters on a daily basis. I'm certainly not going to deny that, the first times, I felt insecure.” (Interview 3, male, surgery, first cycle)

### Sharing mentor

Three mentors (2 female, 1 male) found it most important to share what it means to be a doctor and work with patients. Necessary competencies such as enthusiasm, being open and empathic were seen in function of that role. These ‘sharing’ mentors wanted to be a role model for their mentees and to guide their development both on a professional and a personal level. They preferred a less stringent structure: they often started with the guidelines, but then deviated more from the schedule and script, depending on the topic of the sessions. Two of them were in their second cycle.

### Advising mentor

The other 3 mentors (2 male, 1 female) in this study could be categorized as ‘advising’ mentors. Their focus was more on giving information and advice on both personal, practical and professional matters. They allowed the mentee to take the initiative and gave advice when needed. ‘Advising’ mentors found the intervision techniques too artificial, constrictive and classroom-like. They mentioned fewer competencies and had few expectations of the outcome. They had doubts about the usefulness of the mentoring programme or believed that mentoring competencies cannot be taught. Two of them were in their second cycle.

## Discussion

In this study, we examined how mentors perceived and performed their mentor roles and tried to meet expectations in a longitudinal mentoring programme where reflection is stimulated in the training and the scripts with guidelines for every session. 

All 16 mentors in this study emphasized the importance of respect and trust and the participation of each student. In other mentoring studies trust and respect were perceived as the major prerequisites for open reflection and discussion of meaningful challenges.^27^^,^^28^ In a longitudinal programme, the mentoring relationship can evolve over several years; hence, it can facilitate openness and reflective discourses.^29^

The concept of mentoring in the Ghent University programme of medicine aims at ‘reflective’ mentors and 10 out of 16 mentors clearly represented this profile. Even these mentors struggled in varying degrees with the application of the intervision model of reflection, mostly because of varying degrees of prior experience with reflection. They grew in their role with support of the training with other mentors and the scripts with guidelines. Heeneman and de Grave stress the importance of an active mentor community that supports the development of mentoring skills, especially for novice mentors.^30^ Three mentors still partially met the programme objectives with more emphasis on ‘sharing’ what it means to be a doctor. The ‘sharing’ profile of the 2 mentors in their second cycle may be partially attributed to their self-confidence in their mentoring role and their familiarity with the curriculum and training. It allowed them to be more directly responsive to students' expectations and to share their experience of what it is to be a doctor with less emphasis on the reflective method. It is well established that roles can alternate and change throughout the course of mentoring.^19^^,^^20^^,^^31^ The 3 ‘advising’ mentors did not meet the objectives. A known main pitfall of mentoring is that mentors offer solutions instead of enabling students to find their own solutions and support that process.^28^^,^^32^ As in our study, these mentors often express doubts about the effectiveness of the mentoring programme.^19^ Overall, only 4 of the 8 second cycle mentors were categorized as ‘reflective’. Only 2 out of 5 surgeons were ‘reflective’. A possible explanation is that the contact with patients in general practice or in non-surgical disciplines lends itself better to a reflective attitude.

The 3 profiles that resulted from our analysis have many similarities with mentoring positions in the studies of Stenfors-Hayes and colleagues and Loosveld and colleagues.^19^^,^^20^ Mentors ‘who listen and stimulate reflection’^19^ or ‘monitors’^20^ aim to assist the mentees in becoming reflective learners to empower them to make their own decisions. A mentor ‘who shares what it is to be a doctor/dentist’^19^ or a ‘coach’^20^ encourages mentees to think about their development towards meeting the challenges of their personal and future professional lives. Mentors ‘who answer questions and give advice’^19^ or ‘facilitators’^20^ see themselves as sources of information and are willing to share it by giving advice and answering questions. These profiles may overlap in individual mentors. In these two studies participants were selected in different undergraduate programmes: medicine and dentistry,^19^ medicine, biomedical sciences and health sciences.^20^ It was unclear which guidelines they received in the mentor training.

Our study suggests that the training of mentors can be better adapted to the different profiles of mentors and according to the level of experience with reflection in their professional life. It is also important that experienced mentors keep coming to training sessions to both share their valuable expertise and reinforce their own training. Training sessions should enable mentors to reflect on their beliefs and mentoring style. Loosveld and colleagues argued that not only the ‘how’ of mentoring should be covered, but also the implicit knowledge and beliefs fundamental to the mentoring practice.^33^ Mentor training should continue to clarify the purpose and objectives of reflective mentoring, as mentors grow in their mentoring. As shown in previous studies, inconsistencies regarding the purpose of reflection are a significant barrier to the facilitation of reflective practice.^1^^,^^12^^,^^27^^,^^34^^,^^35^ Reflection is equally important for mentors as a means to grow and develop both personally and professionally.^15^

One caveat though, the way reflection has been operationalized into a teachable and measurable construct could lead to a ‘reflective zombie’: students who have been conditioned to follow prescribed thought steps rather than engaging in truly reflective behaviour.^36^ Still De la Croix and Veen argue there might be an initial phase where reflection starts as a form of acting and gradually becomes more authentic.^36^ Many of our mentors saw their students clearly grow in their reflective role throughout the years. To foster effective reflection, they should not strictly adhere to the guidelines but stimulate the awareness of the personal reflection style of mentees as part of the reflective process. This step is necessary if reflection is not to be a mandatory exercise, both for mentees and mentors, but a gateway to lifelong learning that physicians are able and motivated to continue into their professional practice.^36^

Our findings that 3 out of 16 mentors did not comply with the objectives of the mentoring programme, also raise the question of the need for mentor selection. Hardly any research has been done on selection criteria for medical mentors. The only selection criterion to become a mentor in this programme was being a physician with a commitment to invest time, energy, and effort in mentoring students according to the programme objectives. Mentoring competencies suggested in literature are very broad and dependent on the objectives of the mentoring programme.^4^^,^^32^^,^^34^ Assessment scales for mentors have been used in various medical settings.^37^^-^^39^ Both mentor beliefs and assessment scales may be useful instruments in selection of mentors, depending again on the objectives of the programme.

### Strengths and limitations

We selected the group of mentors in this Belgian study by purposeful sampling. Within this small group, there is a large diversity of specialisations and an equal division between men and women and first and second cycle mentors.  It is important to keep in mind that our selection process may not reflect the full diversity of over 200 mentors. Our research was conducted with a sample population at a single academic institution and therefore we remain cautious regarding transferability.

### Implications for future research

This qualitative study contributes to further insights in the role of reflection in mentoring, the importance of mentor beliefs and the effects of mentor training. These findings can be useful when developing mentoring programmes and implementing mentor selection and training in medical and other health professions, even though the goals and objectives of mentoring are dependent on the specific curriculum. More studies are needed to understand how reflection is stimulated and integrated in mentoring programmes and especially on the effects of reflection in mentoring on the professional identity formation of mentees. We also raised the question of the need for mentor selection which requires further research on selection criteria and methods.

## Conclusions

The findings of this first Belgian study about medical mentorship indicate that, even in a reflection-based programme, the way mentors perceive their role has a profound effect on how and whether reflection is taught to medical students. These differences may have an impact on their professional identity formation and on their growth process in becoming a reflective and compassionate physician.

Training sessions should enable mentors to reflect on their beliefs and mentoring style to evaluate and adapt their practice. The training of mentors can be better adapted according to the level of experience with reflection in their professional life. Experienced mentors need to be motivated to attend training sessions to both share and improve their skills. Mentors should stimulate the awareness of a mentee’s personal reflection style as part of the reflective process.

This study also raises the question of the need for mentor selection. Mentor beliefs and assessment scales may be useful tools in selection of mentors, depending on the objectives of the mentoring programme.

### Acknowledgements

The authors thank all participating mentors and Prof. Peter Pype, Prof. Dominique Benoit and Prof. Jürgen Pieters for valuable feedback on the final draft of this article.

No funding was received for the conducting of this study.

### Conflict of Interest

The author declares that there is no conflict of interest.

## Supplementary material

Supplementary file 1 Appendix. Interview guide
