# Supplementary material for: How do mentors perceive and perform their role in a reflection-based mentoring programme for medical students?
Source: Int J Med Educ. 2024 Oct 28;15:130–8. doi: 10.5116/ijme.6712.35da (PMC11687378; doi:10.5116/ijme.6712.35da)
Supplement: Supplementary file 1 — Appendix. Interview guide [file ijme-15-130-S1.pdf]

Appendix

Interview guide

Motivation

- What was your motivation to become a mentor?
  - If second cycle of mentoring: what was your motivation to continue being a mentor?

Role and relationship

- How do you perceive your role as a mentor?
  - How do you perform this role within the mentoring sessions?
- According to you, what are the main tasks of the mentor within the mentoring programme?
- As a mentor, what do you want to accomplish with the students?
  - How do you try to accomplish this?
  - To what extent do you think your attempts have been successful?
- As a mentor, is there anything you want to accomplish for yourself?
  - Do you experience any benefits of being a mentor?

Competencies and training

- As a mentor, which competencies do you need to perform your mentoring role and tasks?
  - In what way have your competencies developed?
  - When second cycle mentoring: are there things you do differently compared to your first cycle?
- How can the necessary competencies and skills be supported by the mentor training?
  - In the current mentor training, to what extent do you feel the necessary competencies and skills are already addressed?
  - Do you have any suggestions for improvement?
- What are the dos and don’ts
  - As a mentor?
  - During a mentoring session?

Group mentoring and one-on-one mentoring

- What are possible advantages of group mentoring?
- What are possible disadvantages of group mentoring?
- As a mentor, how do you perceive the relationship between mentor and mentee?
  - Do you experience any differences in this relationship during the group sessions and the individual session? If yes, which differences do you experience?
- What are your thoughts on group mentoring versus one-on-one mentoring?

Extra

- Is there anything you would like to add? Any thoughts, advice,…regarding the mentoring programme?
